# Stretch speed‐dependent myofiber damage and functional deficits in rat skeletal muscle induced by lengthening contraction

**DOI:** 10.14814/phy2.12213

**Published:** 2014-11-20

**Authors:** Tomohiro Mori, Nobuhide Agata, Yuta Itoh, Masumi Miyazu‐Inoue, Masahiro Sokabe, Toru Taguchi, Keisuke Kawakami

**Affiliations:** 1 Physical and Occupational Therapy Program, Nagoya University Graduate School of Medicine, Nagoya, Japan; 2 Department of Rehabilitation, Nagoya University Hospital, Nagoya, Japan; 3 Aichi Medical College for Physical and Occupational Therapy, Kiyosu, Japan; 4 Mechanobiology Laboratory, Nagoya University Graduate School of Medicine, Nagoya, Japan; 5 Department of Neuroscience II, Research Institute of Environmental Medicine, Nagoya University, Nagoya, Japan

**Keywords:** Evans blue dye, lengthening contraction, stretch speed

## Abstract

Exercise involving lengthening contraction (LC) often results in delayed myofiber damage and functional deficits over the ensuing days. The present study examined whether the stretch speed of LC is a determinant of damage severity. Under isoflurane anesthesia, LC was repeatedly induced in rat ankle extensor muscles at different stretch speeds (angular velocities of 50, 100, 200, and 400 deg/sec) over a fixed stretch range of motion (90°). The number of muscle fibers labeled with Evans blue dye, a marker of muscle fiber damage associated with increased membrane permeability, increased with the angular velocity of LC (by 20% of all myofibers at 400 deg/sec). Muscle fibers with cross‐sectional areas in the range of 3600–4800 *μ*m^2^, corresponding to type IIb fiber size, exhibited the most severe damage as revealed by the largest decrease in the number of fibers 3 days after LC at 200 deg/sec, suggesting that muscle damage occurred preferentially in type IIb myofibers. Isometric torque of dorsiflexion measured 2 days after LC decreased progressively with LC angular velocity (by 68% reduction at 400 deg/sec). The angular velocity of muscle stretch during LC is thus a critical determinant of the degree of damage, and LC appears to damage type IIb fibers preferentially, resulting in a disproportionate reduction in isometric torque. This LC response is an important consideration for the design of physical conditioning and rehabilitation regimens.

## Abstract (precis)

Representative triple immunofluorescent images of sections from tibialis anterior 2 days after lengthening contraction at different angular velocities. The number of Evans Blue Dye ‐positive fibers increases with the angular velocity of lengthening contraction.

## Introduction

Strenuous exercise can cause structural damage to muscles (Friden and Lieber 1992), resulting in a reduced capacity to produce and sustain force (Armstrong 1984; Proske and Allen 2005). Lengthening contraction (LC), a type of muscle contraction in which the muscle is contracted while it is being stretched, is known to cause greater damage after exercise compared with other types of muscle contraction, such as shortening or isometric contraction.

One possible determinant of this LC‐associated damage is the stretch speed of LC. In rats, LC induced the release of creatine kinase from muscle in a stretch speed‐dependent manner (Warren et al. 1993). Faster LC in human subjects resulted in greater force reduction and a higher elevation in plasma creatine kinase compared with slower LC (Chapman et al. 2006). However, the severity of muscle injury appeared unrelated to the velocity of stretch in rats and mice (Lynch and Faulkner 1998; Brooks and Faulkner 2001). These experiments were conducted in situ and ex vivo using linear stretch, which may not adequately model LC during exercise or reflect the changes that occur in vivo following LC.

Structural changes in the muscle following LC have been documented by both light microscopy (Armstrong et al. 1983) and electron‐microscopy (Newham et al. 1983; Ogilvie et al. 1988). However, quantification in these studies was difficult due to the wide variety of changes. Furthermore, lack of randomization when choosing the area for muscle analysis may have introduced sampling bias. Hamer et al. (2002) first introduced Evans blue dye (EBD) as a marker of increased myofiber membrane permeability (indicative of cell damage) to study the effects of LC. Evans blue staining appeared highly sensitive to subtle membrane changes not detected by standard histological techniques such as hematoxylin–eosin (H–E) staining. Moreover, myofibers were stained by EBD in an all‐or‐none fashion with few weakly labeled fibers, greatly aiding analysis (Hamer et al. 2002). Thus, histological changes indicative of muscle damage can be quantified by counting EBD‐positive cells. However, precise resolution of the time course of damage is not feasible using EBD because nearly a day is needed to obtain maximum labeling.

In the present study, we used a customized device that enables precise control of stretch parameters during LC to investigate whether stretch speed is a critical determinant of muscle fiber damage and functional deficits in rat muscle.

## Methods

### Ethical approval

The present study was conducted according to the Regulations for Animal Experiments in Nagoya University and the Fundamental Guidelines for Proper Conduct of Animal Experiments and Related Activities in Academic Research Institutions in Japan.

### Animals

Sixty‐five male Wistar rats (8 weeks old, 232–265 g; SLC Inc., Shizuoka, Japan) were used for immunohistochemistry and muscle function tests. Animals were housed two per cage in a clean air‐conditioned room (set temperature: 23°C) under a 12 h light/dark cycle (lights on 08:00–20:00 h) with free access to food and filtered clean water throughout the experiment. At the end of the muscle function test, the animals were euthanized by cervical dislocation for immunohistochemistry.

### Lengthening contraction

**Figure 1.**
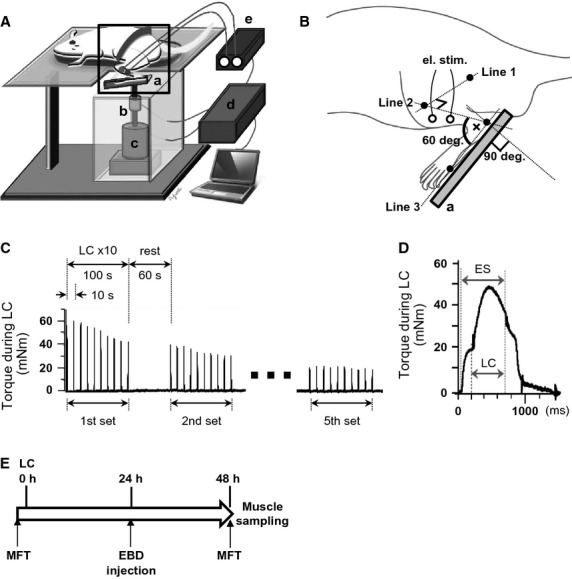
Device and parameters for lengthening contraction (LC). (A) Device and experimental setup for loading LC. The animal is placed on a table under inhalation anesthesia with 1.5% isoflurane. Repetitive electrical stimulation of the tibialis anterior (TA) via surface electrodes induces dorsiflexion of a paw attached to a foot plate (a). The planta pedis was double‐stick taped on the foot plate of the device, and the foot and plate was further wrapped with a heat shrinkable tube. The extensors are repetitively stretched by a stepping motor (c) driven by signals from a computer via a control box (d). These stretch loads are synchronized with electrical stimulation (e) (and thus with dorsiflexion) for LC. A sensor (b) detects the torque generated during LC. (B) An enlarged image of the square area in A. Before triggering LC, the ankle joint was passively moved and positioned at the starting position (30° of dorsiflexion, i.e., the angle formed where the Line 2 and 3 cross each other is 60°). Line 2 connects 2 bony landmarks, the head of the fibula and lateral malleolus, and Line 3 connects the lateral malleolus and the head of the 5th metatarsal bone. The axis of joint rotation is 3 mm in front of the lateral malleolus of the fibula. The ankle joint was plantarflexed (stretched) to 90° from the starting position at angular velocities of 50, 100, 200, and 400 deg/sec while the ankle extensor muscles were contracted by electrical stimulation. (C) Schedule for applying LC and change in torque generated during LC at an angular velocity of 200 deg/sec. Each LC trail was repeated 10 times every 10 sec for 1 set. Sets were repeated every 60 sec to a total of 5. (D) A torque curve generated by the 5th LC in the first set is shown in (c). ES: electrical stimulation. A delay of 200 msec was set from the start of ES to the onset of torque generation by LC. (E) The time line of this experiment is shown. We produced the model of injured rats (*n* = 23) by lengthening contraction (LC) of the anterior tibial muscle (TA). Evans blue dye was administered 24 h after LC. Forty‐eight hours after LC, the maximum isometric dorsiflexion torque of the ankle joint was measured (MFT, muscle function test) and calculated the rate of change from torque value for preinjury. TA muscle was finally removed 48 h after LC for histological analyses.

Under inhalation anesthesia with 1.5% isoflurane, ankle extensor muscles were subjected to repetitive LC using a customized device (NDH‐1; Bio Research Center, Co., Ltd., Nagoya, Japan) that precisely controls stretch parameters (Fig. 1A). Left dorsiflexor muscles of the hind leg were percutaneously stimulated via a pair of surface electrodes fixed with a quick‐drying glue over the tibialis anterior (TA) muscle (Fig. 1B), and maximal dorsiflexion was evoked using supramaximal tetanic current (1 msec pulses at 100 Hz, constant current of 5 mA) supplied through an isolator (SS‐202J; Nihon Kohden Corp., Japan) connected to an electrical stimulator (SEN‐3301; Nihon Kohden Corp., Tokyo, Japan.). The movement of a foot plate was synchronized with the electrical stimulator so that the muscles were stretched while they were being activated. Three axes were drawn so that the range of motion during LC could be precisely defined: the femoral axis (Line 1 between the third trochanter and lateral condyle of the femur), fibular axis (Line 2 between the head and lateral malleolus of the fibula) and foot axis (Line 3 along the base of metatarsal; Fig. 1B). Based on these axes, knee, and ankle joints were set at 90° and 60°, respectively, just before triggering LC. The center of the moment arm of the ankle joint during LC was set 3 mm in front of lateral malleolus of the fibula. LC was induced at angular velocities of 50, 100, 200, and 400 deg/sec with simultaneous stimulus trains of 2000, 1100, 650, and 425 msec, respectively. There was a 200 msec delay from the start of electrical stimulation to the onset of applied torque generation for LC (Fig. 1D, marked by the left thick dotted vertical line). Lengthening contraction was applied over 90° from the starting position (ankle joint dorsiflexed 30°). One LC set was composed of 10 contractions every 10 sec. Five sets were acquired per animal at 60 sec intervals (Fig. 1C). Torque generated in response to electrical stimuli was continuously recorded during LC (Fig. 1C and D), and the peak value was measured at different angular velocities.

### Immunohistochemistry

To assess the integrity of the muscle fiber membrane after LC, animals received an intraperitoneal injection of 1% (wt/vol) EBD (14220‐52; Kanto Chemical Co., Inc, Tokyo, Japan) in PBS (pH 7.4) at a volume of 1% of body mass (BM) (1 mg EBD/0.1 mL PBS/10 g BM). This solution was administered 24 h after LC and 24 h before muscle isolation and histology to obtain the maximum fluorescence signal 2 days after LC (Fig. 1E) as previously reported (Hamer et al. 2002). EBD binds to serum albumin and passes across the damaged sarcolemma into cells from the extracellular space (Hamer et al. 2002). The EBD signal was detected by fluorescent microscopy at 568 nm. Two days after LC, animals were anesthetized with 1.5% isoflurane and TA excised from the surrounding tissues between the origin and insertion. The muscle was rinsed in physiological saline, blotted dry using filter paper, weighed, and then snap frozen. Transverse sections from the middle portion of TA were cut at 10 *μ*m thick. Sections were washed in PBS for 10 min, permeabilized in 0.5% Triton/PBS for 10 min, blocked in 1% bovine serum albumin (BSA)/PBS (1 mg/mL) for 1 h, and then incubated in rabbit anti‐laminin polyclonal primary antibody (1:20000, L9393; Sigma–Aldrich, St Louis, MO) for 1 h at room temperature. After washing with 1% BSA/PBS, immunolabeled sections were incubated for 1 h with a goat anti‐rabbit IgG antibody conjugated to Alexa Fluor^®^ 568 (1:400, A11036; Molecular Probes, Rockville, MD). The sections were counterstained with 4′,6‐diamidino‐2‐phenylindole (DAPI, 1:10000, D‐9564; Sigma–Aldrich, St Louis, MO) to visualize muscle nuclei. After rinsing, sections were mounted in 90% glycerol/PBS. To quantify the number of EBD‐positive fibers, images of cross‐sectional areas (CSAs) of the muscle belly were acquired by fluorescence microscopy (Nano Zoomer RS 2.0; Hamamatsu Photonics, Shizuoka, Japan) and counted all the myofiber numbers in the CSA of the muscle belly using ImageJ software (National Institutes of Health, Bethesda, MD). A muscle fiber intracellularly labeled with EBD and labeled along the cell membrane by anti‐laminin antibody was considered “positive” independent of the amount of dye in the cell. CSA of TA at the center of the belly was measured from fluorescent images by ImageJ software.

We examined if LC preferentially damages a specific class of myofiber because the EBD signals could be seen preferentially in cells with the larger size. First, we measured CSA of all stained myofibers by drawing the outline of the cell membranes in sections immunostained for dystrophin, a protein specifically located at the cell membrane. In brief, 3 days after LC at an angular velocity of 200 deg/sec, animals were anesthetized and the TA muscle sections were prepared as described. Sections were stained with anti‐dystrophin rabbit polyclonal primary antibody (1:400, SC‐15376; Santa Cruz Biotechnology, Santa Cruz, CA) for 1 h at room temperature. Visualization of dystrophin immunostaining and nuclear counterstaining were performed as described above. Naïve animals without LC served as a control. Second, the size of specific fiber subclasses (type I, IIa, and IIb) was measured in a similar way from naïve rats without LC. Successive sections prepared as described were immunostained with myosin heavy chain (MHC) slow monoclonal primary antibody (1:40, VP‐M67; Vector Laboratories, Burlingame, CA), mouse anti‐MHC IIa mouse monoclonal primary antibody (1:40, SC‐71; Developmental Studies Hybridoma Bank (DSHB), Iowa, IA) or anti‐MHC IIb mouse monoclonal primary antibody (1:40, BF‐F3; DSHB) and immunolabeling visualized using Alexa Fluor^®^ 568 goat anti‐rabbit IgG (1:400, A11036; Molecular Probes), Alexa Fluor^®^ 488 goat anti‐mouse IgG (1:400, A11029; Molecular Probes) or Alexa Fluor^®^ 488 goat anti‐mouse IgM (1:400, A21042; Molecular Probes), respectively. CSA of each muscle fiber was measured using ImageJ. Each staining pattern was examined in six sections from six rats.

### Muscle function test

Under inhalation anesthesia with 1.5% isoflurane, isometric dorsiflexion torque was measured just before and 2 days after LC with the same device (NDH‐1; Bio Research Center, Co., Ltd.) used for loading LC (Fig. 1E). Dorsiflexor muscles were percutaneously stimulated via a pair of surface electrodes placed over TA and maximal dorsiflexion was evoked using supramaximal tetanic current (650 msec train duration, 1 msec pulses at 100 Hz, constant current of 5 mA).

### Statistical analyses

Results are expressed as mean ± SEM. Group means from immunohistochemistry and muscle function tests were compared by one‐way analysis of variance (ANOVA) followed by Tukey's post hoc test for pairwise comparisons. The correlations between the number of EBD‐positive fibers and isometric torque generated during dorsiflexion after LC and between the peak torque generated during LC and the angular velocity were analyzed by Spearman's test. Interaction between muscle fiber size and treatment (LC angular velocity) was analyzed by two‐way repeated measures ANOVA followed by Sidak's multiple comparison test. *P *<**0.05 was considered significant.

## Results

### Stretch speed‐dependent muscle damage

**Figure 2.**
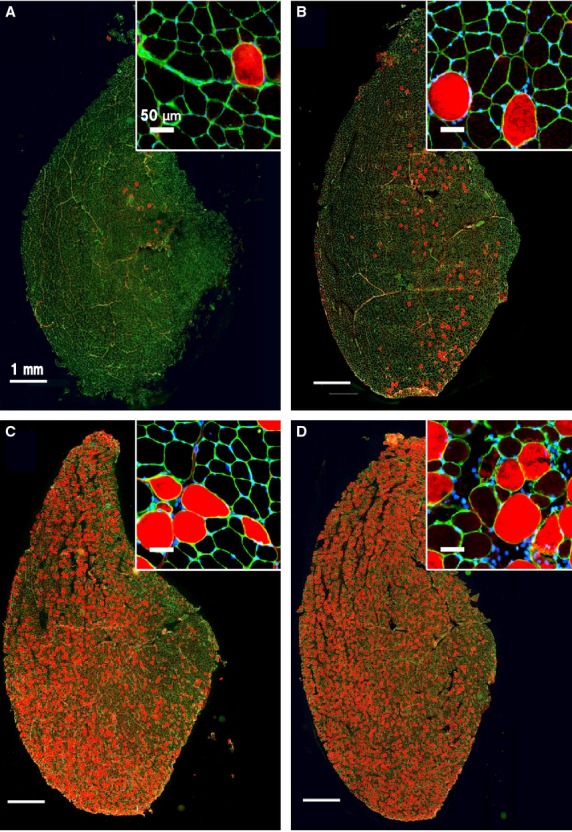
Stretch speed‐dependent damage of muscle fibers. Representative triple immunofluorescent images of sections from tibialis anterior (TA) 2 days after lengthening contraction (LC) at different angular velocities: 50 deg/sec (A), 100 deg/sec (B), 200 deg/sec (C), and 400 deg/sec (D). Red: damaged myofibers stained with Evans blue dye (EBD), green: muscle cell membrane labeled with anti‐laminin antibody, blue: nuclei of muscle cells stained with 4′,6‐diamidino‐2‐phenylindole (DAPI). Scale bar: 1 mm. Anterior side of TA; left and medial side; upper. Enlarged image shown in each inset (scale bar: 50 *μ*m). The number of EBD‐positive fibers increases with the angular velocity of LC and larger‐diameter fibers are preferentially labeled in an all‐or‐none fashion.

To examine whether stretch speed affects the severity of LC‐induced muscle damage, rats were subjected to varying LC angular velocities (50, 100, 200, and 400 deg/sec) during electrically evoked ankle extensor muscle contraction. This was followed by in vivo EBD staining and subsequent analysis of EBD‐positive myofiber numbers (as an index of muscle damage or increased sarcolemma permeability) in triple immunofluorescence‐labeled sections of TA. The number of EBD‐positive fibers appeared to increase with LC stretch speed (Fig. 2), and dye labeling was observed preferentially in relatively large‐diameter cells in a roughly all‐or‐none fashion. In contrast, there were no EBD‐positive cells in TA sections from untreated rats (*n* = 6, photograph not shown).

**Figure 3.**
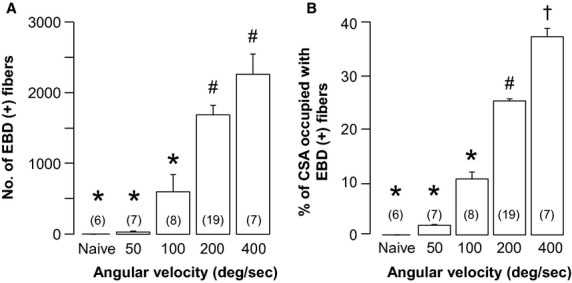
Stretch speed‐dependent muscle damage. (A) Number of damaged muscle fibers labeled with Evans blue dye (EBD) in transverse sections of tibialis anterior (TA) 2 days after lengthening contraction (LC). The number of EBD‐positive fibers in the entire cross‐sectional area (CSA) of TA increases with angular velocity of muscle stretch (*n* = 7, 8, 19 and 7 at angular velocities of 50, 100, 200, and 400 deg/sec, respectively). (B) The proportion of CSA occupied by EBD‐positive fibers increases with LC angular velocity (*n* = 7, 8, 19, and 7 at angular velocities of 50, 100, 200, and 400 deg/sec, respectively). Data are shown as mean ± SEM. Values with different symbols above bars are significantly different.

The number of EBD‐positive cells within the entire CSA of the TA belly increased progressively with angular velocity (50 deg/sec: 31.9 ± 14.0 cells, *n* = 7; 100 deg/sec: 598.8 ± 241.4 cells, *n* = 8; 200 deg/sec: 1677.2 ± 133.2 cells, *n* = 19; 400 deg/sec: 2252.6 ± 285.8 cells, *n* = 7; Fig. 3A). The number of labeled cells was significantly higher in TA sections from animals subjected to LC at 200 deg/sec or 400 deg/sec compared with 100 deg/sec or 50 deg/sec (*P* < 0.05, one‐way ANOVA followed by Tukey's post hoc test). The mean total number of myofibers in the entire CSA of the TA counted in 1 rat following LC at an angular velocity of 200 deg/sec was 11487. The proportion of the total CSA of the TA belly occupied by EBD‐positive fibers also increased progressively with angular velocity (50 deg/sec: 1.8% ± 0.1%, *n* = 7; 100 deg/sec: 10.4% ± 1.3%, *n* = 8; 200 deg/sec: 25.0% ± 0.4%, *n* = 19; 400 deg/sec: 37.0% ± 1.6%, *n* = 7; Fig. 3B) and was significantly higher in sections from animals subjected to LC at 200 deg/sec or 400 deg/sec compared with 100 deg/sec or 50 deg/sec (*P* < 0.05, one‐way ANOVA followed by Tukey's post hoc test).

Both total TA wet weight and CSA of transverse sections taken from around the center of the muscle belly also increased progressively with LC stretch speed (Table 1), consistent with muscle damage and edema.

**Table 1.** Stretch speed‐dependent edematous changes in the muscle after LC

	Naive	Angular velocity (deg/sec)			
		50	100	200	400
Muscle wet weight (mg)	471.8 ± 19.1 (6)	431.8 ± 9.0 (7)	479.4 ± 12.5 (8)	494.0 ± 11.0 (19)^**^	521.6 ± 17.8 (7) ^***^
Muscle belly CSA (mm^2^)	33.2 ± 2.2 (6)	32.1 ± 0.8 (7)	37.9 ± 1.6 (8)	38.1 ± 1.1 (19)^**^	43.2 ± 1.7 (7) ^***^^,^^*^

Tibialis anterior (TA) muscle samples were obtained from rats 2 days after lengthening contraction (LC). Data are presented as mean ± SEM. Wet weight and cross‐sectional area (CSA) of TA increased in parallel with the LC angular velocity.

*P < 0.01 versus naive, **P < 0.05 and ***P < 0.01 versus 50 deg/sec, one‐way ANOVA followed by Tukey's post hoc test.

### LC‐induced muscle damage occurred preferentially in type IIb myofibers

**Figure 4.**
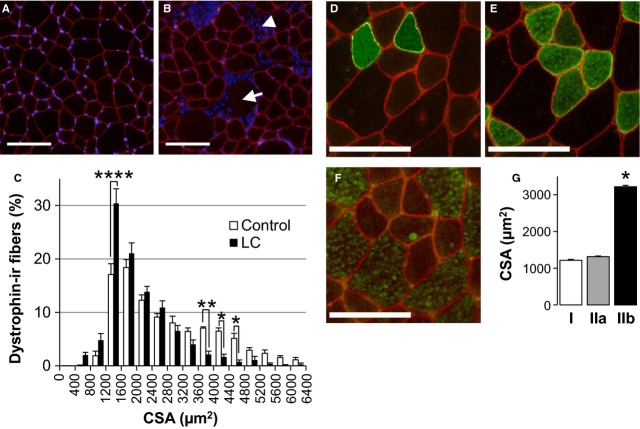
Lengthening contraction preferentially damages type IIb fibers. (A, B) Immunofluorescent images of cross‐sectional areas (CSAs) of tibialis anterior (TA) from naïve rats (A) and the lengthening contraction (LC) group 3 days after LC at an angular velocity of 200 deg/sec. (B) Red: Muscle cell membrane labeled with dystrophin antibody. Blue: 4′,6‐diamidino‐2‐phenylindole (DAPI)‐stained nuclei in muscle cells. Note necrotic fibers without dystrophin immunoreactivity (arrow) and nuclear accumulation (arrow head). Scale bar = 100 *μ*m. (C) The proportion of dystrophin‐immunoreactive myofibers against cross‐sectional area (CSA) of myofibers (Bin width: 400 *μ*m^2^). Open bar: Naïve control (*n* = 6), Filled bar: LC (*n* = 6). Note significant differences between the 2 groups in cells with CSAs in the ranges of 1200–1600 *μ*m^2^, 3600–4000 *μ*m^2^, 4000–4400 *μ*m^2^, and 4400–4800 *μ*m^2^ (*P* < 0.05–0.0001, two‐way repeated measures ANOVA followed by Sidak's test). (D–F) Immunofluorescent images of different myofiber types in TA sections from a naive animal. Red: Muscle cell membrane labeled with dystrophin antibody. Green: Myosin heavy chain‐positive myofibers: type I (D), IIa (E) and IIb (F). Scale bar = 100 *μ*m. (G) Mean CSAs of myofibers from naive animal. Type IIb fibers (*n* = 6) are significantly larger than type I (*n* = 6) and IIa (*n* = 6) fibers (*P < 0.05, one‐way ANOVA followed by Tukey's post hoc test).

The EBD labeling was observed predominantly in larger‐diameter myofibers, suggesting that these larger fibers are preferentially damaged by LC. We labeled cells with an antibody against dystrophin, a cell membrane protein, to measure the CSA distribution of myofibers in naïve rats and rats subjected to LC at 200 deg/sec (Fig. 4A and B). The size distribution was markedly altered by LC, particularly for fibers with CSA >3200 *μ*m^2^. Two‐way repeated measures ANOVA revealed a significant size group (LC speed) interaction (*F*[15, 150] = 9.08, *P* < 0.0001), consistent with differential sensitivity of specific fiber classes to LC. Although the effect of the group was not significant (*F*[1, 10] = 0.14, *P* > 0.05), there were significant differences between the control (*n* = 6) and the combined LC group (*n* = 6) for fibers with CSAs within the ranges 1200–1600 *μ*m^2^, 3600–4000 *μ*m^2^, 4000–4400 *μ*m^2^, and 4400–4800 *μ*m^2^ (*P* < 0.05–0.0001, Sidak's test, Fig. 4C). To gain a rough estimate of the myofiber size range preferentially damaged, we examined the relationship between the size (CSA) and myofiber types in naive animals. CSA ranges of type I fibers (1212.5 ± 141.3 *μ*m^2^, *n* = 6, a sample photo in Fig. 4D) and IIa fibers (1327.5 ± 146.5 *μ*m^2^, *n* = 6, a sample in Fig. 4E) were less than a half that of the type IIb fibers (3238.1 ± 231.6 *μ*m^2^, *n* = 6, a sample in Fig. 4F; *P* < 0.05, one‐way ANOVA followed by Tukey's post hoc test, Fig. 4G). Given that the largest decrease occurred in fibers >3200 *μ*m^2^ in CSA, LC appears to preferentially damage type IIb fibers.

### Stretch speed‐dependent decrease in muscle function

**Figure 5.**
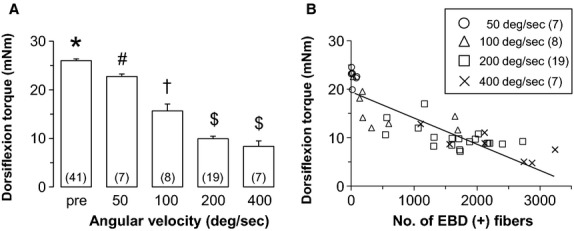
Stretch speed‐dependent loss of muscle function. (A) Evoked torque of dorsiflexion induced by isometric contraction of the ankle extensor muscles measured 2 days after lengthening contraction (LC). (A) Torque decreases as a function of increased angular velocity from 50 to 400 deg/sec. Data are shown as mean ± SEM. Number of animals tested is shown in parenthesis in each bar. Bars with different symbols indicate significant differences. Number of samples pre‐LC (*n* = 41). (B) Correlation between the number of Evans blue dye (EBD)‐positive myofibers and the isometric dorsiflexion torque generated during LC. Note the significant negative correlation between the two parameters (Spearman *r* = −0.85, *P* < 0.0001).

Concomitant with fiber damage, we also observed functional deficits following LC. The isometric torque (generated by electrically induced dorsiflexion of ankle extensors) measured 2 days after LC was significantly lower compared with the pre‐LC baseline (26.0 ± 0.4 mNm, *n* = 41, Fig. 5A) and decreased progressively with LC speed (50 deg/sec: 22.7 ± 0.5 mNm, *n* = 7, 13% decrease from baseline; 100 deg/sec: 15.7 ± 1.4 mNm, *n* = 8, 40% decrease; 200 deg/sec: 9.9 ± 0.5 mNm, *n* = 19; 62% decrease; 400 deg/sec: 8.4 ± 1.1 mNm, *n* = 7; 68% decrease). The loss in torque was not different between the 200 deg/sec and 400 deg/sec treatment groups. There was a significant negative correlation between the number of EBD‐positive myofibers in the entire CSA of TA and the isometric dorsiflexion torque measured 2 days after LC (Spearman *r* = −0.85, *P* < 0.0001, Fig. 5B), strongly suggesting that functional loss is a direct result of fiber damage.

The peak torque generated during LC increased as a function of angular velocity (Table 2), and there was a weak but significant correlation between the peak torque and LC angular velocity (*n* = 41, Spearman *r* = 0.382, *P* < 0.05). The peak torque differed significantly between 50 deg/sec and 400 deg/sec treatment groups (one‐way ANOVA followed by Tukey's post hoc test).

**Table 2.** Stretch speed‐dependent increase in peak torque generated during lengthening contraction (LC)

Angular velocity (deg/sec)	50	100	200	400
No. of animals tested	7	8	19	7
Peak torque (mNm)	42.1 ± 2.1	44.0 ± 6.6	45.9 ± 3.2	48.9 ± 1.6*

Peak torque generated during LC increased in parallel with LC angular velocity. Data are presented as mean ± SEM.

* *P* < 0.05 compared with 50 deg/sec, one‐way ANOVA followed by Tukey's post hoc test.

## Discussion

### Stretch speed‐dependent muscle damage

In the present study, we demonstrate that the degree of muscle damage following LC, as indicated by the number of EBD‐permeable myofibers, the expanded CSA of the muscle belly and muscle weight gain, increases progressively with the angular velocity of LC. These undesirable changes may result from excessive tension generated in the muscle during LC because mechanical factors are considered important for initiation of muscle damage and loss of function triggered by LC (McCully and Faulkner 1986; Warren et al. 1993; Friden and Lieber 2001). Indeed, the peak torque generated during LC increased with angular velocity of LC but then decreased over subsequent days.

The magnitude of the observed structural damage was directly related to the stretch speed of LC (and the peak torque generated during LC), as reported previously by (McCully and Faulkner 1986). In their study, the contracting muscles of mice were lengthened in situ by 20% of fiber length (*L*_f_) at 0.2, 0.5, and 1.0 *L*_f_/sec and histological signs of injury (e.g., infiltration of inflammatory cells) were evident at 0.5 and 1.0 *L*_f_/sec but not at 0.2 *L*_f_/sec. In our case the number of EBD‐positive damaged cells significantly increased after LC at 200 deg/sec while the number at 50 deg/sec and 100 deg/sec was not significantly different from the one in naïve animals. Thus, the threshold for muscle damage appeared to be between 100 deg/sec and 200 deg/sec in our study (rat TA) and between 0.2 and 0.5 *L*_f_/sec in mouse extensor digitorum longus (McCully and Faulkner 1986). Both studies found that peak torque generated during LC increased with LC stretch speed. According to our rough estimates, the rat extensor muscles were stretched by 16% of *L*_f_ at speeds of 0.5, 1.1, 2.2, and 4.4 *L*_f_/sec (corresponding to 50, 100, 200, and 400 deg/sec, respectively). In the present study, changes at 50 deg/sec and 100 deg/sec, which correspond closely to 0.5 and 1.0 *L*_f_/sec in their study (McCully and Faulkner 1986), should result in total muscle stretch of 16%, less than the 20% *L*_f_ used by McCully and Faulkner (1986). The difference in damage suggests that extension length is also a contributing factoring to LC‐induced muscle damage, but this issue requires further study using constant stretch speed while varying *L*_f_. It is difficult to evaluate independently the effect of stretch speed and peak torque with the present experiment setup. However, generally and as presented in the present study, as the stretch speed and concomitant peak torque value have a positive correlation, we can say that the speed‐dependent peak torque may be the direct cause of muscle damage.

### Stretch speed‐dependent decrease in muscle function

The maximal isometric torque generated by dorsiflexion of the ankle joint 2 days after LC decreased progressively as the angular velocity of LC was increased, in accordance with previous findings that a linear muscle stretch in situ decreased isometric force in a stretch speed‐dependent manner (McCully and Faulkner 1986). As the decrease in torque implies a loss of muscle function, the increased stretch speed of LC is a critical determinant of these delayed functional deficits.

The functional deficit was related to the degree of muscle damage as revealed by a strong negative correlation between the two parameters. However, the loss of muscle function induced by LC was large compared with the degree of observable muscle damage. Indeed, isometric torque was reduced by 62% following LC at 200 deg/sec and by 68% after LC at 400 deg/sec, and the torque decreases were associated with only 15% and 20% reductions in fiber numbers. Assuming a total number of muscle cells in TA of 11487 as estimated in our study, the proportional areas occupied by damaged myofibers (i.e., EBD‐positive area) were 25% and 37%, respectively. One possible explanation for this remarkable dissociation between the decrease in peak torque and the proportion of damaged fibers is that the type of muscle fiber damaged contributes disproportionately to force generation. Analysis of the difference in fiber diameter distribution between naïve and LC‐damaged muscles suggested that type IIb fibers, which were estimated to be >2 times larger (by CSA) than type I and IIa fibers, were preferentially damaged. As type IIb fibers can indeed generate greater tension compared with other fiber types, the preferential damage to type IIb fibers resulted in a functional deficit disproportionate to the number of fibers damaged. An alternative explanation; however, is that force generation was also reduced in EBD‐negative (presumably healthy) myofibers. In fact, loss of contractile strength is induced not only by damage observable by light microscopy but also by breakdown of excitation–contraction (E–C) coupling (Balnave and Allen 1995; Morgan and Allen 1999; Warren et al. 2002). This E–C uncoupling may occur as a result of damage to the cell membrane, physical disruption of the tension‐bearing elements in myofibrils, a shift in the optimum length by overstretching sarcomeres and local contracture, and microscopic injury to the sarcoplasmic reticulum. Muscle swelling (as indicated by increased wet weight and CSA) may also contribute to the decrease in tension, although it is unclear whether this is a direct factor.

## Conclusion

We demonstrate that LC leads to muscle damage and functional deficits that are dependent on the LC stretch speed. If this is also true for humans, this effect must be considered when designing exercise programs to improve fitness or for rehabilitation. The customized device that we developed for the present study enabled precise control of exercise parameters, thereby providing a means for studying quantitative changes in histology and muscle function at both the gross and cellular levels. This device could prove useful for future studies in sports medicine and rehabilitation.

## Acknowledgments

We are deeply grateful to Mizumura whose comments and suggestions were innumerably valuable throughout the course of our study.

## Conflict of Interest

The authors declare that there were no conflicts of interest related to the study.
